# Amino acid mutations PB1-V719M and PA-N444D combined with PB2-627K contribute to the pathogenicity of H7N9 in mice

**DOI:** 10.1186/s13567-024-01342-6

**Published:** 2024-07-05

**Authors:** Xiaoquan Wang, Xin-en Tang, Huafen Zheng, Ruyi Gao, Xiaolong Lu, Wenhao Yang, Le Zhou, Yu Chen, Min Gu, Jiao Hu, Xiaowen Liu, Shunlin Hu, Kaituo Liu, Xiufan Liu

**Affiliations:** 1 College of Veterinary Medicine, Yangzhou University, Yangzhou, 225009 China; 2 Joint International Research Laboratory of Agriculture and Agri-Product Safety, The Ministry of Education of China, Yangzhou University, Yangzhou, 225009 China; 3 Jiangsu Co-innovation Center for Prevention and Control of Important Animal Infectious Diseases and Zoonosis, Yangzhou University, Yangzhou, 225009 China; 4 Jiangsu Key Laboratory of Zoonosis, Yangzhou, 225009 China; 5 Yangzhou Center for Disease Control and Prevention, Yangzhou, 225009 China

**Keywords:** H7N9, pathogenicity, virulence factors, vRNP

## Abstract

H7N9 subtype avian influenza viruses (AIVs) cause 1567 human infections and have high mortality, posing a significant threat to public health. Previously, we reported that two avian-derived H7N9 isolates (A/chicken/Eastern China/JTC4/2013 and A/chicken/Eastern China/JTC11/2013) exhibit different pathogenicities in mice. To understand the genetic basis for the differences in virulence, we constructed a series of mutant viruses based on reverse genetics. We found that the PB2-E627K mutation alone was not sufficient to increase the virulence of H7N9 in mice, despite its ability to enhance polymerase activity in mammalian cells. However, combinations with PB1-V719M and/or PA-N444D mutations significantly enhanced H7N9 virulence. Additionally, these combined mutations augmented polymerase activity, thereby intensifying virus replication, inflammatory cytokine expression, and lung injury, ultimately increasing pathogenicity in mice. Overall, this study revealed that virulence in H7N9 is a polygenic trait and identified novel virulence-related residues (PB2-627K combined with PB1-719M and/or PA-444D) in viral ribonucleoprotein (vRNP) complexes. These findings provide new insights into the molecular mechanisms underlying AIV pathogenesis in mammals, with implications for pandemic preparedness and intervention strategies.

**Supplementary Information:**

The online version contains supplementary material available at 10.1186/s13567-024-01342-6.

## Introduction

As members of the Orthomyxoviridae family of viruses, influenza A viruses (IAVs) have eight-segmented, negative, single-stranded RNA genomes. In accordance with the antigenic specificity of hemagglutinin (HA) and neuraminidase (NA) proteins on the surface of IAVs, they can be categorized into subtypes H1–H18 and N1–N11, respectively. Except for subtypes H17N10 and H18N11, which are exclusively found in bats [1, 2], all other HA and NA subtype strains primarily originate from wild birds and poultry and are collectively referred to as avian influenza viruses (AIVs) [3]. During more than a century of evolution since the first report of AIVs in Italy in 1878, AIVs have gradually been able to cross interspecies barriers, infecting a wide range of mammals, including humans [3–5].

Multiple subtypes of AIV can infect humans, including H5N1/6, H7N4/9, H10N3/8, H3N8 and H9N2 [6–13]. Among them, H7N9 subtype AIVs cause the greatest number of human infections (1568 cases) and are associated with a high fatality rate (39%), posing a serious threat to public health [14]. In this regard, exploring the mechanisms that enhance the infectivity and pathogenicity of H7N9 in mammals is important.

AIVs typically exhibit low pathogenicity in mice, although certain strains can unexpectedly become highly pathogenic without the need for prior adaptation [15]. As we reported previously, two avian-derived H7N9 isolates, namely, A/chicken/Eastern China/JTC4/2013 (JTC4) and A/chicken/Eastern China/JTC11/2013 (JTC11), are genetically highly similar but exhibit different pathogenicities in mice [16]. The five proteins encoded by the JTC4 and JTC11 viruses differ by only six amino acids located in five different proteins, i.e., polymerase basic protein 2 (PB2), PB1, polymerase acid protein (PA), HA and NA. To understand the genetic basis for the different virulence levels of the two H7N9 viruses in mice, we generated a series of mutation viruses using reverse genetics. Our study revealed that the synergy among polymerase proteins is the pivotal molecular mechanism influencing the virulence of H7N9 in mice.

## Materials and methods

### Viruses and cell lines

The H7N9-subtype AIVs A/Chicken/Eastern China/JTC4/2013 and A/Chicken/Eastern China/JTC11/2013 were obtained from chickens and preserved in our laboratory [16]. The viruses were amplified in 9-day-old specific-pathogen-free (SPF) embryonated eggs (Merial, China) and stored at −80 °C. Primary chicken embryo fibroblasts (CEFs) were prepared in our laboratory. Human embryonic kidney 293 (HEK293T), Madin-Darby canine kidney (MDCK), and human lung carcinoma (A549) cells were grown in Dulbecco’s modified Eagle’s medium (DMEM; Invitrogen, USA) supplemented with 10% fetal bovine serum (FBS; Gibco, New Zealand).

### Plasmid construction and viral rescue

All gene segments of A/Chicken/Eastern China/JTC4/2013 and A/Chicken/Eastern China/JTC11/2013 were cloned and inserted into the pHW2000 vector, as previously described [17]. Eight plasmids were co-transfected into HEK293T cells using Lipofectamine 2000 Reagent (Invitrogen, USA). Culture supernatant aliquots were harvested at 72 h post-transfection, and 9-day-old chicken embryos were infected and incubated for 96 h. The parental and reassorted viruses were purified for three rounds and confirmed by sequencing to ensure the absence of unwanted mutations. Aliquots of viral fluid were stored at −80 °C.

### Mouse challenge experiments

Six-week-old female BALB/c mice (Yangzhou Experimental Animal Center, Jiangsu, China) were used as animal models to investigate the pathogenicity of these mutated viruses to mammals. Groups of five mice were anaesthetized with Zoletil® 50 (tiletamine-zolazepam (Virbac, Carros, France), 20 mg/g body weight) and intranasally (i.n.) inoculated with 50 µL of virus in tenfold serial dilutions from 10^3.0^ to 10^6.0^ 50% egg infective dose (EID_50_). To determine the mouse lethal dose at 50% (MLD_50_), all mice were monitored daily for 14 days, and death was recorded on a daily basis. Humane euthanasia (overdose CO_2_) was used for mice with a body weight loss >25% of their initial body weight. In accordance with Reed and Muench, the MLD_50_ values were calculated [18]. In each group, three mice were euthanized 3 days post-infection (dpi) and samples from the nasal turbinates, lungs, kidneys, and brain were collected for virus titrations.

The lungs of mice infected with the indicated viruses at 3 dpi were harvested, fixed in 10% neutral-buffered formalin, embedded in paraffin, and sliced into 4 µm sections. Hematoxylin–eosin (H&E) staining was performed on the sections. Lung tissue sections were scored based on pathological changes: 0, no visible lesions; 1, area affected by the lesions (<10%); 2, area affected by the lesions (<30%, ≥10%); 3, area affected by the lesions (<50%, ≥30%); and 4, area affected by the lesions (≥50%). We measured lung edema based on the ratio of wet to dry weight of the lungs of virus-inoculated or control mice. The lungs were then heated to 65 °C in a gravity convection oven for 24 h and weighed to obtain the dry mass. The wet/dry weight ratio was calculated by dividing the dry mass by the wet mass.

Quantitative real-time PCR (qRT‒PCR) was used to qualitatively assess the levels of Mx1, interferon-beta (IFN‒β), interleukin-beta (IL-1β), IL-6, inducible protein-10 (IP‒10) and interferon-stimulated gene 15 (ISG15) in lung homogenates. In brief, the lung homogenates were collected at 3 dpi, RNA was extracted and converted into cDNA, and then the levels of cytokine expression were analysed with TB Green premix Ex TaqTM II on a LightCycler 480 System instrument. The expression of glyceraldehyde-3-phosphate dehydrogenase (GAPDH) was also detected and used to normalize gene expression between samples.

### Viral replication in different host cell lines

To evaluate growth dynamics in vitro in different cell lines, MDCK, A549 and CEF cells were grown on 6-well plates and infected with the indicated viruses at a multiplicity of infection (MOI) of 0.001 and a 50% tissue culture infectious dose (TCID_50_). Following 1 h of incubation, the viruses were removed, and the cells were washed three times with PBS. The cells were incubated at 37 °C with essential medium containing 0.5% BSA. At 12, 24, 36, 48, 60 and 72 h post-infection (hpi), virus-containing culture supernatants were harvested. Viral titres are expressed as the TCID_50_/mL in MDCK cells.

### Polymerase activity assay

Viral polymerase activity was determined using a luciferase reporting assay. Viral ribonucleoprotein (vRNP) complexes composed of PB2, PB1, PA and nucleoprotein (NP) derived from JTC4 and JTC11 were separately cloned and inserted into the pCAGGS vector. HEK293T cells were co-transfected with PB2, PB1, PA, and NP expression plasmids (200 ng) together with the firefly luciferase reporter plasmid p‐Luci (300 ng) and the internal control Renilla plasmid (30 ng). At 24 h, the cell lysates were processed to measure firefly and Renilla luciferase activities using a GluMax 96 microplate luminometer (Promega).

### Statistical analysis

We used GraphPad Prism 9 for graphing and statistical analyses (GraphPad Software, San Diego, CA, USA). To make one-way comparisons between multiple groups, one-way ANOVA was used, and two-way ANOVA was used to make two-way comparisons. Statistical significance was reported at *P* < 0.05, *P < 0.05, **P < 0.01, ***P < 0.001, and ****P < 0.0001.

## Results

### Polymerase proteins cooperatively increased H7N9 virulence in mice

**Figure 1:**
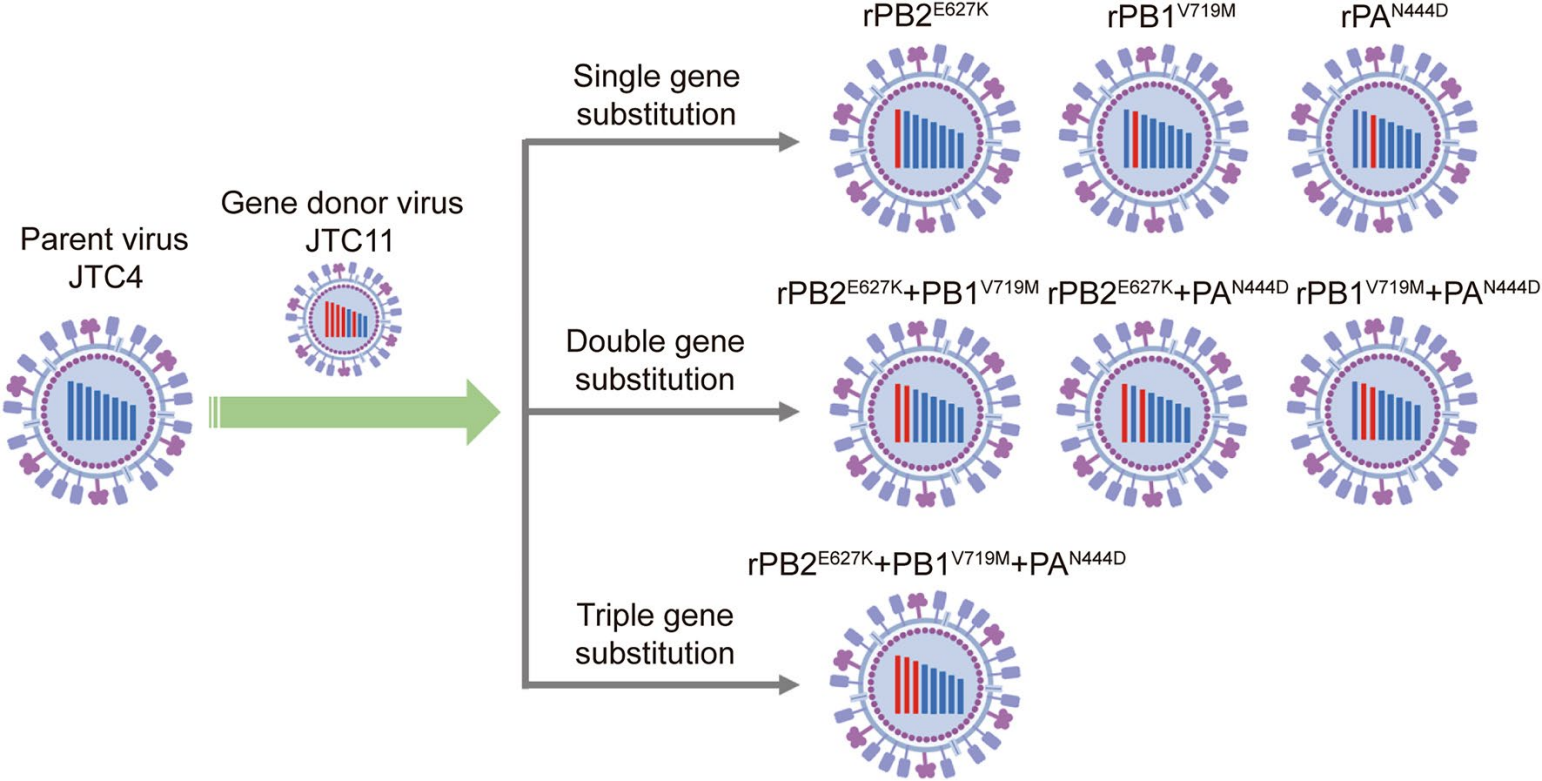
**Schematic composition of the gene replacement of H7N9 viruses.** The eight gene segments of the virus are, from left to right, polymerase B2 (PB2), polymerase B1 (PB1), polymerase A (PA), hemagglutinin (HA), nucleoprotein (NP), neuraminidase (NA), matrix protein (M), and non-structural protein (NS). Blue represents JTC4, while red indicates the genes differing between JTC11 and JTC4.

The JTC4 and JTC11 viruses differ by only six amino acids located in five different proteins, i.e., PB2, PB1, PA, HA and NA. While the pathogenicity and host-range specificity of AIVs are influenced by multiple genetic factors, the polymerase protein plays a crucial role in determining these properties [19–21]. To identify amino acids in the polymerase protein of JTC11 that contribute to its high virulence in mice, we compared the PB2, PB1 and PA sequences of JTC4 and JTC11. Only three amino acids (PB2-627E/K, PB1-719V/M and PA-444 N/D) were different [16]. According to previous studies, the PB2-E627K substitution is critical for mammalian adaptation [17, 19], and the other two amino acids have not previously been linked to the pathogenicity of influenza viruses. We therefore generated seven reassortants by reverse genetics using JTC4 as the backbone (Figure 1). We designated these reassortants rPB2^E627K^, rPB1^V719M^, rPA^N444D^, rPB2^E627K^ + PB1^V719M^, rPB2^E627K^ + PA^N444D^, rPB1^V719M^ + PA^N444D^, and rPB2^E627K^ + PB1^V719M^ + PA^N444D^ and tested their replication and virulence in mice.

**Figure 2:**
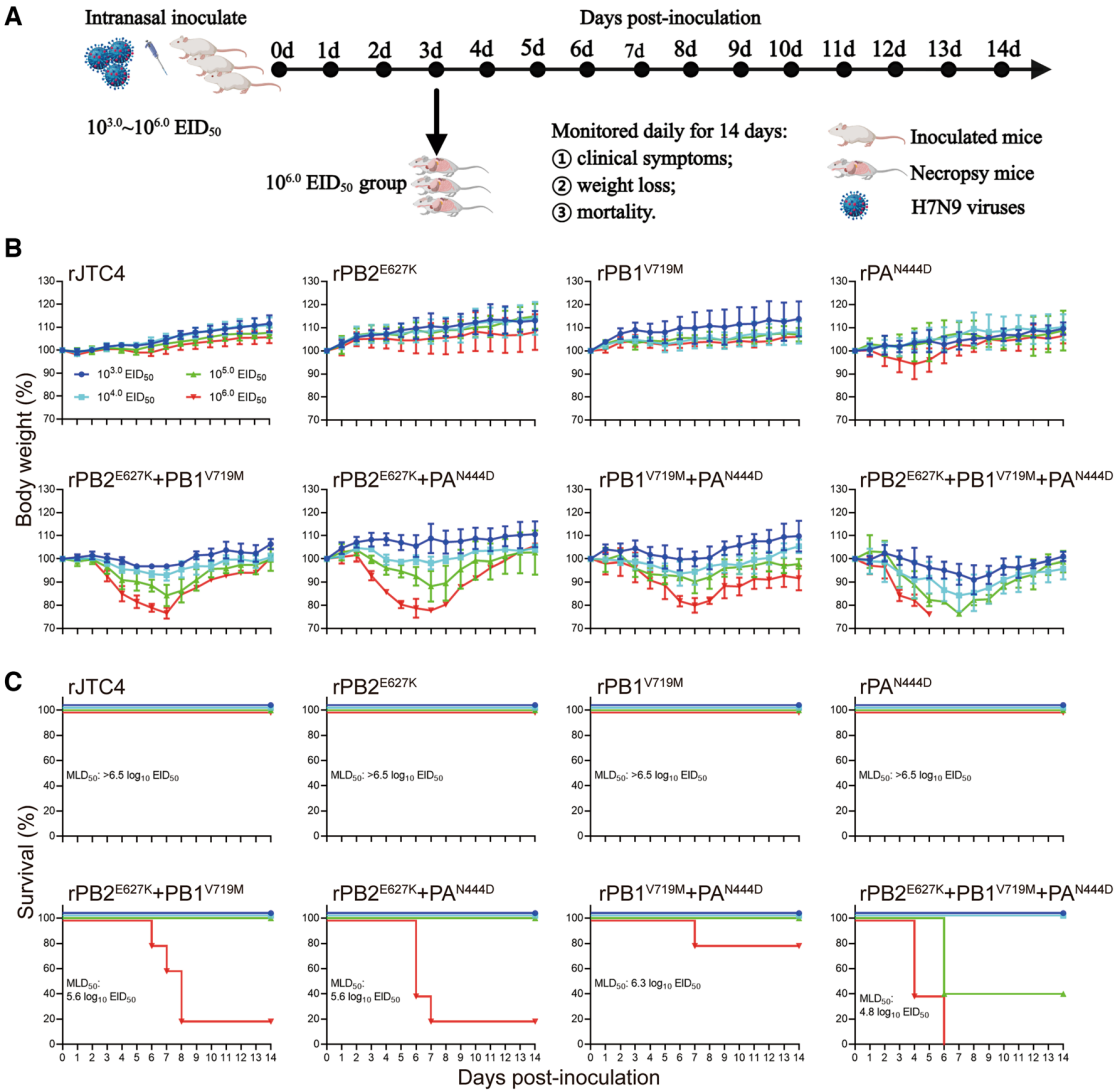
**Mapping of crucial mutation sites in polymerase proteins that increase H7N9 AIV virulence in mice**. **A** Diagram of the experimental procedure. Briefly, groups of 6-week-old five BALB/c mice were inoculated with the indicated viruses at doses of 10^3.0^ ~ 10^6.0^ EID_50_; clinical symptoms, body weight and survival were monitored daily for 14 dpi; mice were humanely killed when they lost ≥ 25% of their initial body weight; and 10^6.0^ EID_50_ group mice were dissected and sampled on day 3 to detect viral load. **B** Body weight loss was monitored for 14 days. **C** Survival was monitored for 14 days.

**Figure 3:**
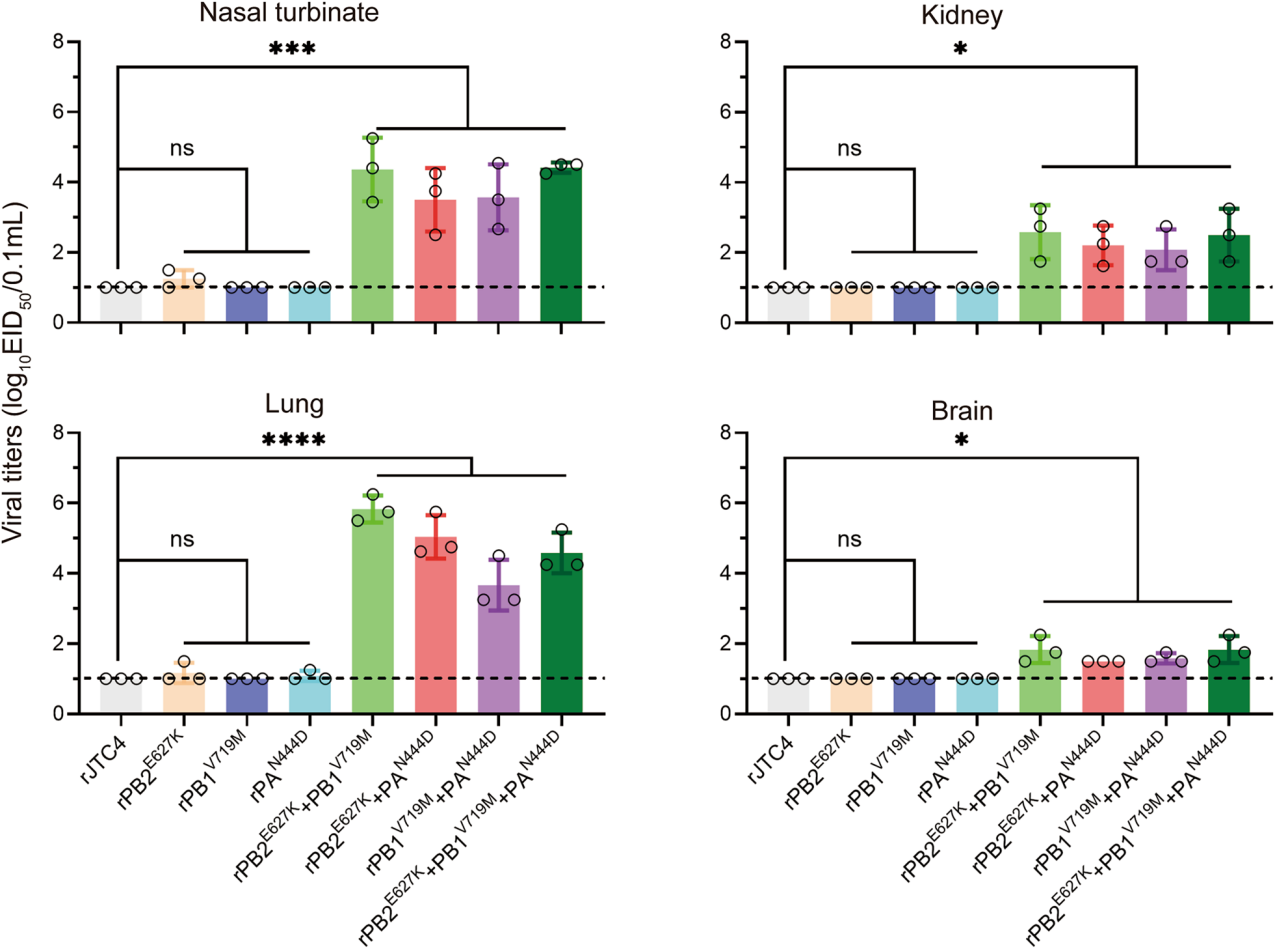
**Polymerase proteins cooperatively increased H7N9 replication in mice.** Mice were challenged with 10^6.0^ EID_50_ of the indicated virus, and three mice per group were euthanized at 3 dpi; virus titres in different organs (turbinate, lung, brain, and kidney) were determined by an EID_50_ assay on chicken eggs; horizontal dashed lines indicate the lower limits of detection.

First, we infected mice with viruses with single mutations in the background of JTC4. As shown in Figure 2, all single-point mutation viruses (rPB2^E627K^, rPB1^V719M^, and rPA^N444D^) caused minor weight loss and no deaths under high-dose infection conditions (10^6.0^ EID_50_), with an MLD_50_ > 6.5 log_10_ EID_50_, indicating that single-point mutations do not affect the pathogenicity of H7N9 in mice. This finding is consistent with the finding that single-point mutation viruses replicate at extremely low titres in multiple organs of mice, even in the lungs (Figure 3). Afterwards, we examined the pathogenicity of the double-point combined mutation strains. As shown in Figure 2, significant weight loss and death were observed in all double-point combined mutation strains (rPB2^E627K^ + PB1^V719M^, rPB2^E627K^ + PA^N444D^, and rPB1^V719M^ + PA^N444D^) under 10^6.0^ EID_50_ infection conditions, with MLD_50_ values of 5.6, 5.6 and 6.3 log_10_ EID_50_, respectively. In addition, these double-point combined mutation strains could increase replication in all organs of mice, even in the brain (Figure 3). These results showed that all double-point synergistic mutations could enhance H7N9 subtype AIV virulence in mice. Additionally, the combined mutant strains of PB2, PB1 and PA (rPB2^E627K^ + PB1^V719M^ + PA^N444D^) further enhanced the virulence of H7N9 in mice (Figure 2), increasing its ability to replicate in various organs (Figure 3). Taken together, the synergy among polymerase proteins plays a crucial role in the heightened pathogenicity of H7N9 in mammals.

### Polymerase proteins cooperatively enhance the ability of H7N9 to induce lung injury and inflammation

**Figure 4:**
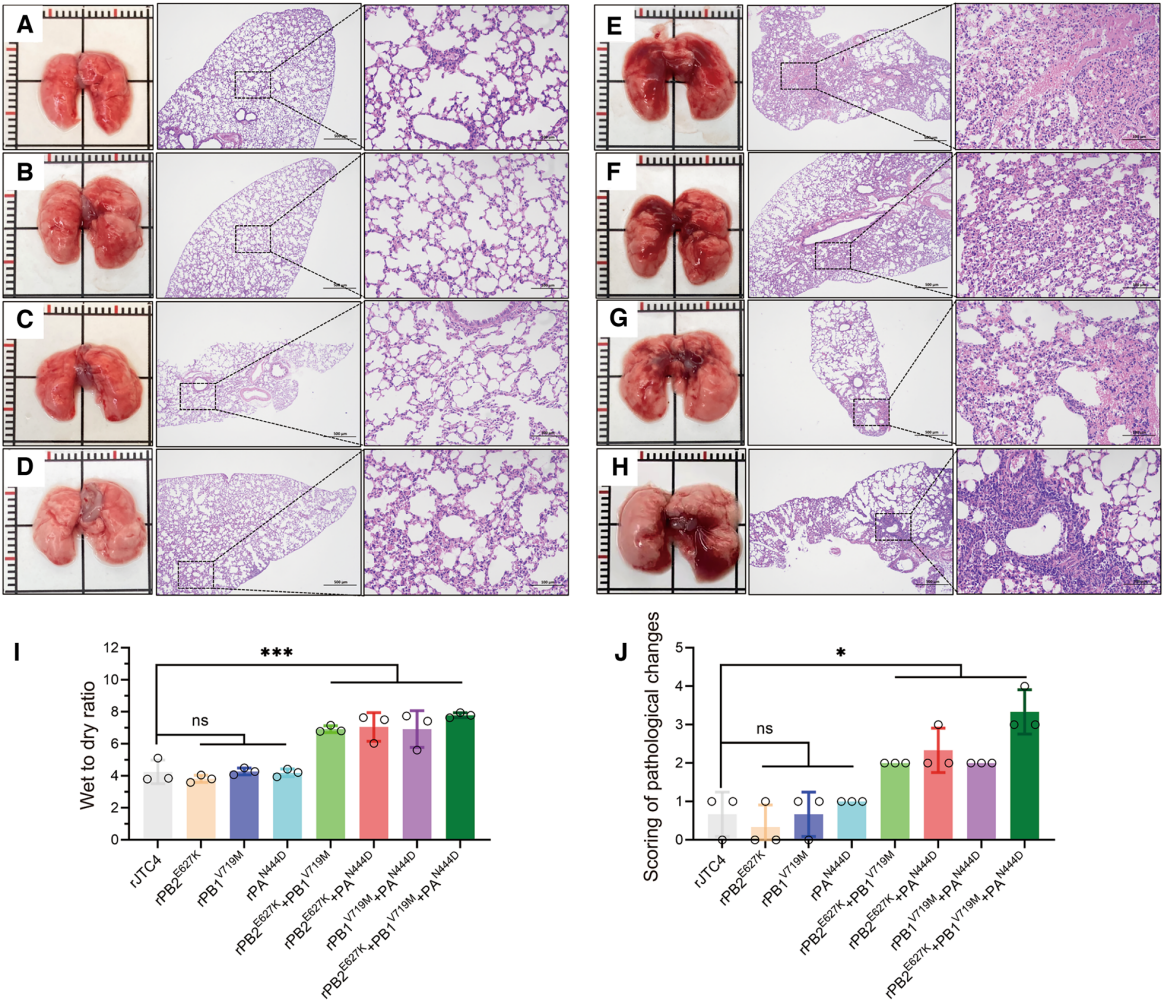
**Polymerase proteins cooperatively increased H7N9-induced lung lesions in mice.** Macroscopic lesions and histopathological (lung sections were stained with H&E) findings in the lungs of mice infected with the indicated viruses at an EID_50_ of 10^6.0^ at 3 dpi. **A**–**H** rJTC4, rPB2^E627K^, rPB1^V719M^, rPA^N444D^, rPB2^E627K^ + PB1^V719M^, rPB2^E627K^ + PA^N444D^, rPB1^V719M^ + PA^N444D^, and rPB2^E627K^ + rPB1^V719M^ + PA^N444D^, respectively. **I** Lung edema was assessed using the lung wet/dry weight ratio. Briefly, the lungs of each virus-inoculated or control mouse were collected and weighed to obtain the wet mass. The lungs were then heated to 65 °C in a gravity convection oven for 24 h and weighed to obtain the dry mass. The wet/dry weight ratio was calculated by dividing the dry mass by the wet mass. **J** Histopathological scores of the lungs of mice inoculated with the indicated viruses. Lung tissue sections were scored based on pathological changes: 0, no visible lesions; 1, area affected by the lesions (<10%); 2, area affected by the lesions (<30%, ≥10%); 3, area affected by the lesions (<50%, ≥30%); and 4, area affected by the lesions (≥50%).

AIVs primarily target the lungs of mammals, resulting in life-threatening lung damage and severe inflammation. After infection with the indicated viruses at an EID_50_ of 10^6.0^, the mice were euthanized on day 3, and the lungs were studied histopathologically. The double-point and three-point combined mutation strains induced pulmonary lesions and focal dark red consolidations (Figures 4E–H). These features were not detected in the lungs of mice inoculated with the single-point mutation viruses (Figures 4B–D). The lungs of the double-point and three-point mutation strain-infected mice showed severe peribronchiolitis as well as bronchopneumonia (thickened alveolar septa, edema, and interstitial inflammatory cells infiltrating the alveoli), whereas mild pathological damage was observed in the lungs of the single mutation viruses (Figure 4). The histopathology scores of the double-point and three-point mutation strain-infected lungs reached 2.0, whereas those of the single-point mutation virus-infected lungs were less than 1.0 (Figure 4J).

**Figure 5:**
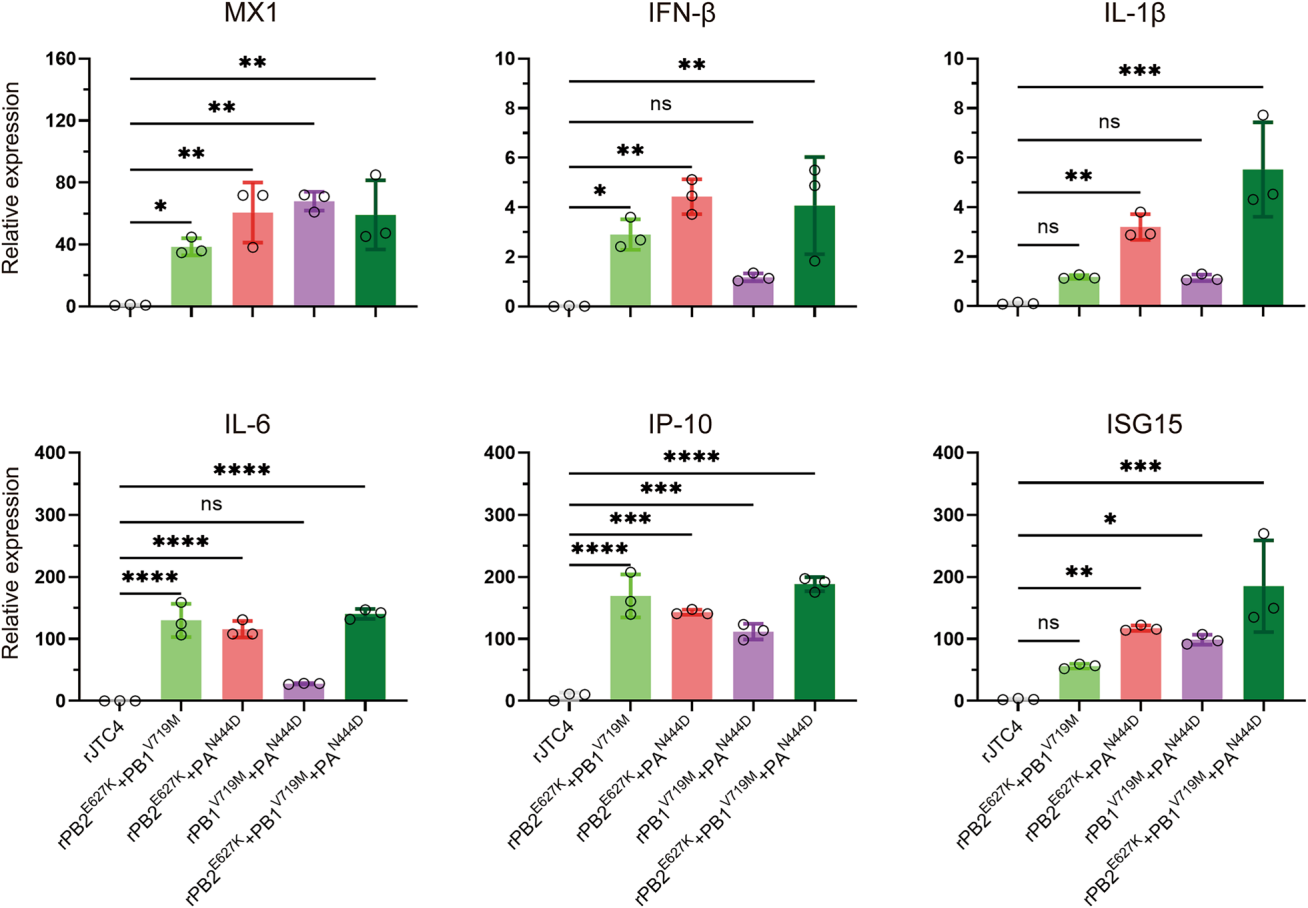
**Inflammatory cytokine expression of the reassortant H7N9 isolates in mice.** Mice were inoculated intranasally with 10^6.0^ EID_50_ of the desired viruses, total RNA was extracted from the lungs at 3 dpi, and equal amounts of RNA were used for qRT‒PCR. The expression level of each cytokine gene was normalized to that of GAPDH and is presented as the fold change compared with the control.

As indicated by the wet-to-dry ratio, the lungs of mice inoculated with the double-point and three-point mutation strains exhibited significant inflammation (Figure 4I). Inflammation is strongly associated with the pathogenicity of AIVs. Afterwards, we evaluated whether mouse lungs were infiltrated with inflammatory cytokines after infection with rJTC4 and single- and multipoint mutation viruses. As shown in Figure 5, the levels of proinflammatory cytokines (IL-1β and IL-6), chemokines (IP-10), and antiviral cytokines (IFN-β, Mx1, and ISG15) were notably elevated in the groups inoculated with multipoint mutation viruses compared to those inoculated with rJTC4. Taken together, these results indicate that polymerase proteins cooperatively enhance the ability of H7N9 to induce lung injury and inflammation.

### Polymerase proteins cooperatively enhance the ability of H7N9 to replicate in mammalian cells

**Figure 6:**
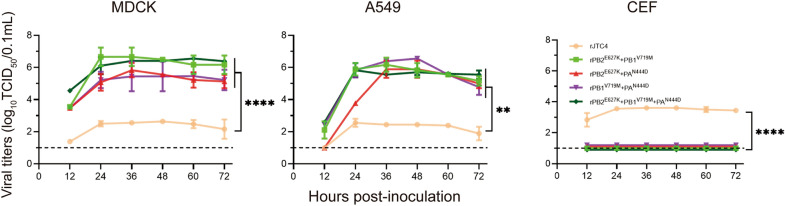
**Growth dynamics of the reassortant H7N9 viruses in different cell lines**. MDCK, A549, and CEF cells were inoculated with the indicated viruses at a multiplicity of infection (MOI) of 0.001 TCID_50_/cell in triplicate at 37 °C. The supernatants were aseptically harvested at 12, 24, 36, 48, 60 and 72 hpi. The virus titres were determined by determining the TCID_50_ per 0.1 mL in MDCK cells.

The replication ability of multipoint mutation viruses was tested in different host cells to mimic host-switching events in vitro. To quantify viral replication dynamics during overt infection (0–72 hpi), MDCK, A549 and CEF cells were inoculated with the indicated viruses at an MOI of 0.001. As shown in Figure 6, multipoint mutation viruses generated significantly higher titres than did the rJTC4 virus during the initial infection stages to late infection stages in mammalian cells (MDCK, A549). Additionally, multipoint mutation viruses lose their ability to replicate in CEFs. According to these results, multipoint mutations in polymerase proteins increase viral replication in mammalian cells and decrease viral replication in CEFs.

### Polymerase proteins cooperatively enhance the polymerase activity of H7N9 in mammalian cells

**Figure 7:**
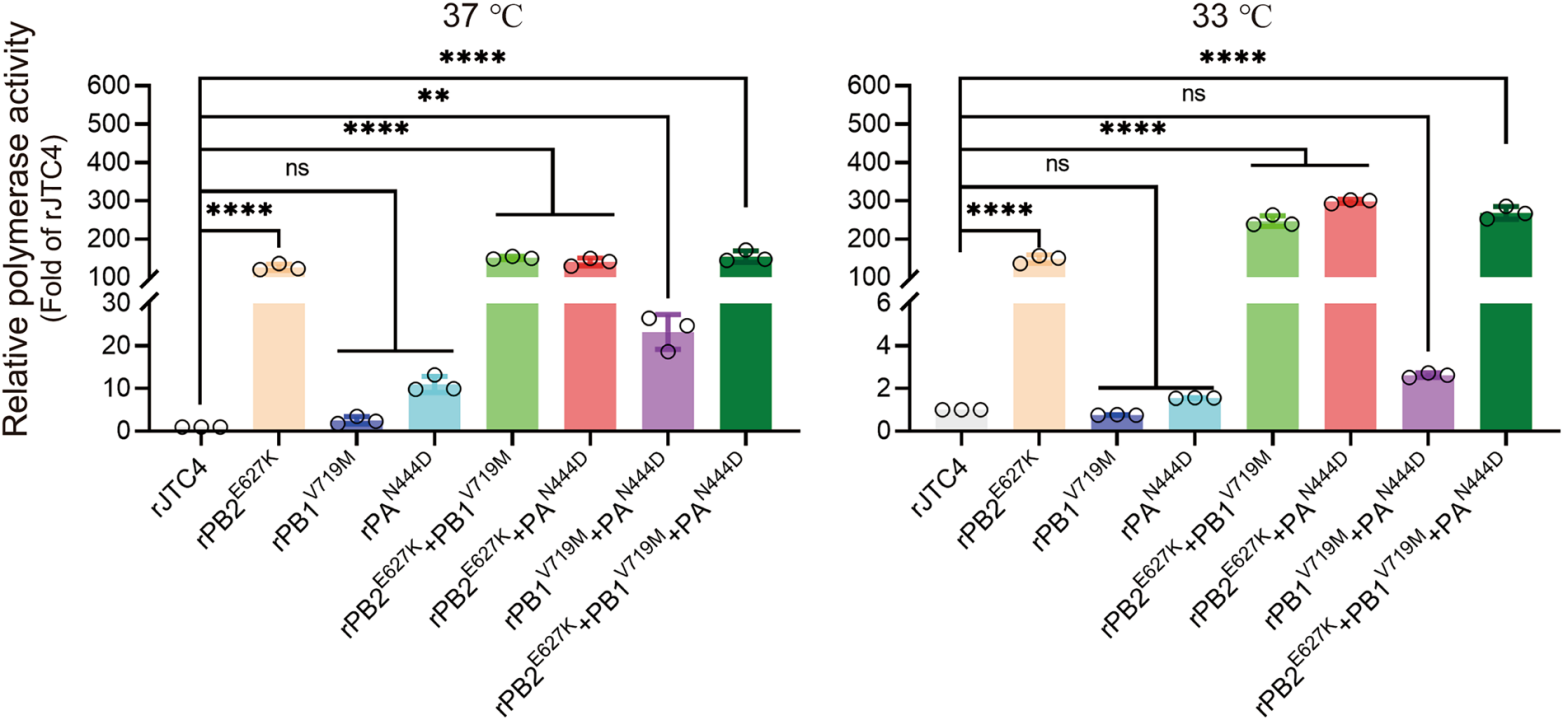
**Polymerase proteins cooperatively increase H7N9 polymerase activity in mammalian cells.** Plasmids encoding the PB2, PB1, PA, and NP proteins derived from JTC4 with the indicated segment substitutions were transfected into HEK293T cells at the indicated temperatures, together with the firefly luciferase reporter plasmid and internal control plasmid. At 24 h post-transfection, luciferase activities were measured.

There is evidence that the viral RNP complex plays a key role in the replication and pathogenicity of AIVs [22, 23]. To ascertain the relative contribution of mutations to RNP polymerase activity, minigenome replication assays were conducted in HEK293T cells at 33 °C and 37 °C (corresponding to the temperatures of the human upper and lower respiratory tracts, respectively [24]). The polymerase activities of rPB2^E627K^, rPB2^E627K^ + PB1^V719M^, rPB2^E627K^ + PA^N444D^ and rPB2^E627K^ + PB1^V719M^ + PA^N444D^ were significantly greater than those of rJTC4 at 33 °C and 37 °C (Figure 7). In addition, the polymerase activity of rPB1^V719M^ + PA^N444D^ was significantly greater than that of rJTC4 at 37 °C but not at 33 °C (Figure 7). These findings suggest that the polymerase activities of H7N9, which possesses PB2-627K alone or PB2-627K combined with PB1-719M and PA-444D, are greater than those of rJTC4 in mammalian cell lines.

### Prevalence of amino acid types at positions PB2-627, PB1-719 and PA-444 in H7N9 viruses

To determine the prevalence of the amino acids PB2-627, PB1-719 and PA-444 in different host-derived H7N9 viruses, available H7N9 PB2, PB1 and PA sequences were downloaded from the Global Initiative on Sharing All Influenza Data (GISAID) and National Center for Biotechnology Information (NCBI) databases [25, 26]. As of December 2023, H7N9 was the subtype with the highest number (1568) of human AIV infection cases. Among these PB2 segments originating from humans, 71.02% carry the E627K mutation (Table 1). In contrast, only 0.9% of PB2 segments originating from avians exhibited the E627K mutation. As shown in Table 1, PB1-719V (99.74%) and 719M (0.13%) were detected in avian-derived H7N9 strains. In addition, there was a slight increase (0.38%) in the proportion of PB1-719M mutations in human-derived H7N9 strains. Similar to PB1-719, PA-444D was also present in natural avian- and human-derived H7N9 strains, and the proportions (0.26% and 0.39%, respectively) were extremely low.

**Table 1:** **Prevalence of amino acid types at positions PB2-627, PB1-719 and PA-444 in H7N9 viruses**

Segment	Site	Amino acid substitutions	Relative frequency (no. of strains with the substitution/total no. of strains)	
			Avian-origin	Human-origin
PB2	627	E	98.77% (882/893)	26.35% (331/1256)
		K	0.9% (8/893)	71.02 (892/1256)
		Others	0.33% (3/893)	2.63% (33/1256)
PB1	719	V	99.74% (754/756)	93.7% (1234/1317)
		M	0.13% (1/756)	0.38% (5/1317)
		Others	0.13% (1/756)	5.92% (78/1317)
PA	444	N	99.74% (755/757)	99.46% (1290/1297)
		D	0.26% (2/757)	0.39% (5/1297)
		Others	0 (0/757)	0.15% (2/1297)

## Discussion

In this study, we explored the genetic basis for the difference in virulence between two avian H7N9 viruses in mice. The JTC11 virus was lethal in mice, with an MLD_50_ of 3.5 log_10_ EID_50_, whereas the JTC4 virus was nonlethal in mice, with an MLD_50_ of > 6.5 log_10_ EID_50_ [16]. Using a reverse genetic system, we found that polymerase proteins are key to H7N9 virulence in mice.

vRNPs contain the viral RNA genome complexed with multiple NP molecules and one RNA-dependent RNA polymerase complex containing PB2, PB1, and PA [27]. The results of homology modelling (Additional file 1) indicated that the PB1-V719M mutation alters the interaction between PB1 and PB2, while the PA-N444D mutation affects the interaction between PA and PB1. These structural changes in the vRNP complex suggest potential alterations in protein function. These vRNPs are the minimal units required for viral transcription and replication [27]. Host restriction factors can block vRNP function, and mammalian AIVs are particularly susceptible to this [28–31]. Therefore, polymerases exhibit limited efficiency in replicating the genome within mammalian cells. AIVs can propagate between mammals, and a high mutation rate in the genome is an important strategy [19, 32–35]. Several host-adaptive mutations in viral polymerase subunits have been identified [20, 21, 23, 36, 37]. The most commonly known adaptive mutation is PB2-E627K: human-adapted AIVs typically encode K, whereas avian AIVs encode E [38, 39]. Notably, several studies have shown that PB2-627K alone can overcome species restriction and increase AIV virulence in mammals [38, 40, 41]. However, in the present study, the PB2-E627K mutation alone was insufficient to increase H7N9 virulence in mice despite its ability to increase polymerase activity in mammalian cells at both 33 °C and 37 °C.

In most cases, host adaptation and pathogenicity of AIVs in mammals are polygenic traits [17, 22, 42, 43]. As we previously reported, we found that hemagglutinin mutations combined with PB2-627K can enhance the pathogenicity and transmissibility of avian H9N2 in mammals [17]. Liang et al. discovered that the emergence of the PB2-E627K mutation in H7N9 AIVs is driven by the intrinsically low polymerase activity associated with the PA protein. This highlights the crucial role of cooperation among polymerase proteins in facilitating AIV adaptation within mammalian hosts [22]. Our results showed that rPB2^E627K^ + PB1^V719M^, rPB2^E627K^ + PA^N444D^, and rPB2^E627K^ + PB1^V719M^ + PA^N444D^ can increase the virulence of H7N9 in mice. Consistent with this, PB1-V719M and/or PA-N444D mutation combined with PB2-E627K further increased polymerase activity in mammalian cells at both 33 °C and 37 °C.

The PB2-E627K mutation first appeared in the 1918 H1N1 pandemic virus, and all human circulating viruses of the twentieth century possessed this signature [44]. H7N9, with 1568 reported human infection cases, stands out as the subtype with the highest incidence. Notably, 71.02% of human-derived viruses harbor the PB2-E627K mutation, underscoring the significance of PB2-627K in AIV adaptation to mammals. In this study, the PB1-V719M and/or PA-N444D mutation combined with PB2-E627K increased virulence in mice. However, the PB1-719M and PA-444D mutations were not prevalent in the current H7N9 strains.

In summary, our findings demonstrate that the cooperation of PB2-E627K, PB1-V719M, and PA-N444D potentially contributes to enhancing the virulence of avian H7N9 in mice. Additionally, we observed that these combined mutations augment polymerase activity, thereby enhancing virus replication, inflammatory cytokine expression, and lung injury, ultimately increasing pathogenicity in mice. Moreover, our study emphasized that AIV virulence is a polygenic trait. The identification of these newly associated virulence-related residues in vRNPs may serve as an early warning for future human pandemics.

### Supplementary Information

**Additional file 1** Effects of mutation on the three-dimensional structure of polymerase proteins.

## Data Availability

The data supporting the conclusions of this article are included within the article. Additional data used and/or analysed during the current study are available from the corresponding author upon reasonable request.
