# Supplementary material for: Amino acid mutations PB1-V719M and PA-N444D combined with PB2-627K contribute to the pathogenicity of H7N9 in mice
Source: Vet Res. 2024 Jul 5;55:86. doi: 10.1186/s13567-024-01342-6 (PMC11227215; doi:10.1186/s13567-024-01342-6)
Supplement: Supplementary file 1 — Additional file 1 Effects of mutation on the three-dimensional structure of polymerase proteins. [file 13567_2024_1342_MOESM1_ESM.doc]

**Additional file 1 Effects of mutation on the three-dimensional structure of polymerase proteins.**

**Method**

In order to analyze the effect of differential amino acids on the polymerase protein on the three-dimensional structure of the protein, we used the homology modeling approach. The polymerase protein sequences of JTC4 and its mutated viruses were compared in the Swiss Model website to obtain polymerase trimer protein models with high homology rates, respectively, and the obtained models were used as templates and analyzed using Pymol software.

**Result**


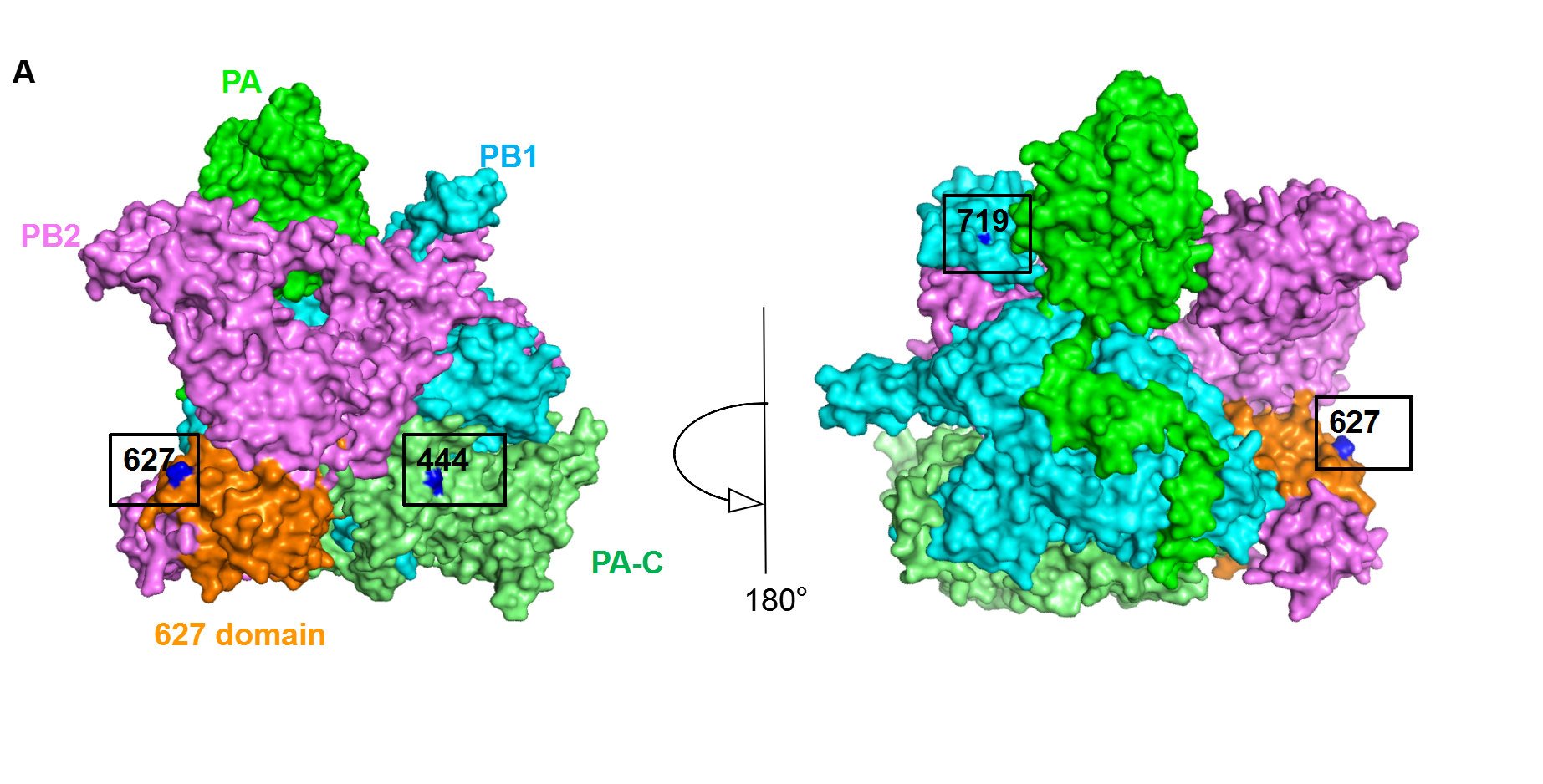

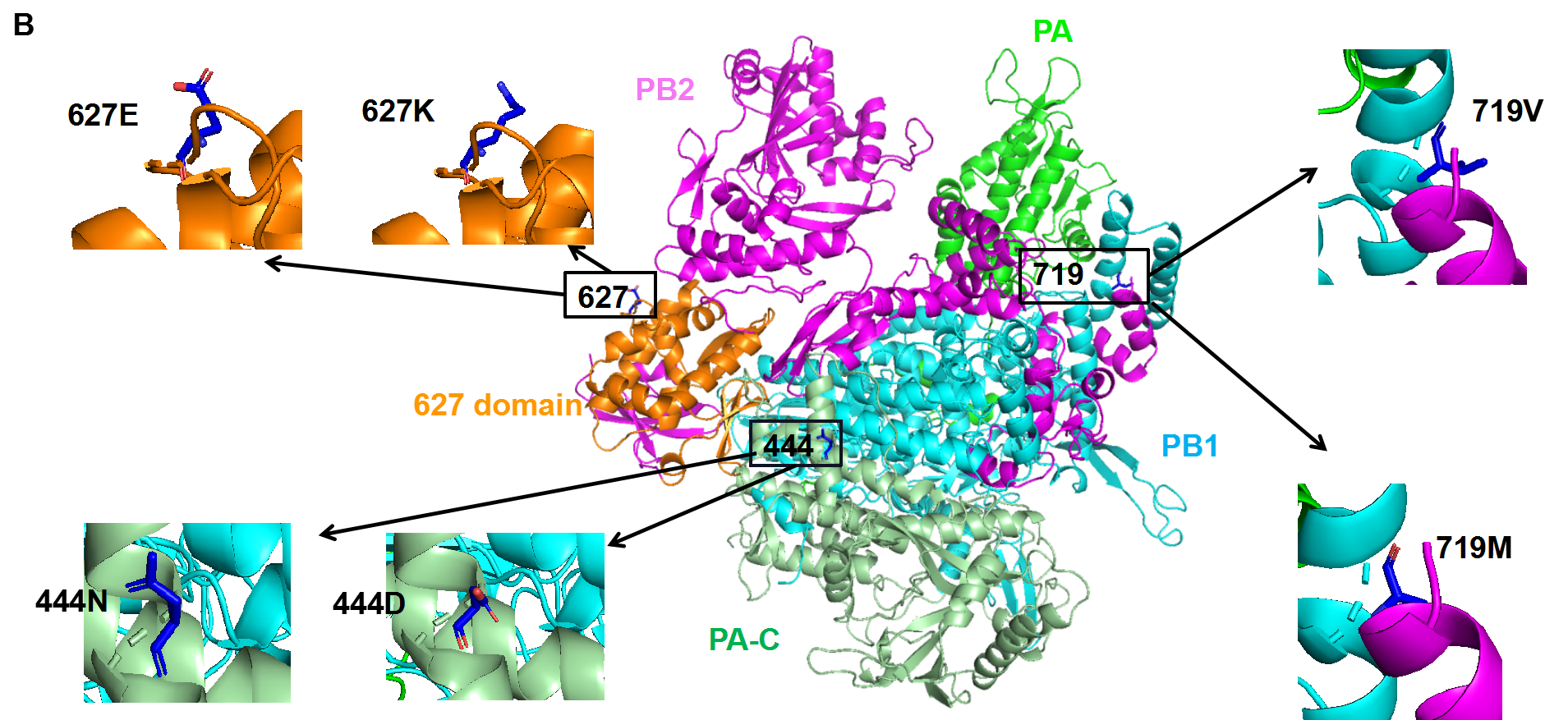
The purpose of this study was to investigate three differential amino acid sites (PB2-E627K, PB1-V719M and PA-N444D) on the polymerase protein, and we predicted the effects of mutations at these sites on the three-dimensional structure of the viral proteins through the Swiss Model website. As can be seen from Additional file 1A, amino acid PB2-627 is located in the 627-domain of PB2 protein, amino acid PA-444 is located on the surface of the C-terminal structural domain of PA protein, and amino acid PB1-719 is located in the C-terminal structural domain of PB1 protein. From Additional file 1B, it can be seen that the mutation of all three sites caused changes in the spatial structure of the protein.
